# “Because I'm Bad at the Game!” A Microanalytic Study of Self Regulated Learning in League of Legends

**DOI:** 10.3389/fpsyg.2021.780234

**Published:** 2021-12-02

**Authors:** Erica Kleinman, Christian Gayle, Magy Seif El-Nasr

**Affiliations:** ^1^Games User Interaction and Intelligence Laboratory, Department of Computational Media, University of California, Santa Cruz, Santa Clara, CA, United States; ^2^Games and Playable Media, Department of Computational Media, University of California, Santa Cruz, Santa Clara, CA, United States

**Keywords:** self-regulated learning, online learning, technology enhanced learning, gamified learning, esports, esports in education, League of Legends

## Abstract

Self-regulated learning (SRL) is a form of learning guided by the student's own meta-cognition, motivation, and strategic action, often in the absence of an educator. The use of SRL processes and skills has been demonstrated across numerous academic and non-academic contexts including athletics. However, manifestation of these processes within esports has not been studied. Similar to traditional athletes, esports players' performance is likely correlated with their ability to engage SRL skills as they train. Thus, the study of SRL in the context of esports would be valuable in supporting players' learning and mastery of play through specialized training and computational support. Further, an understanding of how SRL manifests in esports would highlight new opportunities to use esports in education. Existing work on SRL in games, however, predominantly focuses on educational games. In this work, we aim to take a first step in the study of SRL in esports by replicating Kitsantas and Zimmerman's (2002) volleyball study in the context of League of Legends. We compared the self-regulatory processes of expert, non-expert, and novice League of Legends players, and found that there were significant differences for processes in the forethought phase. We discuss three implications of these findings: what they mean for the development of future computational tools for esports players, implications that esports may be able to teach SRL skills that transfer to academics, and what educational technology can learn from esports to create more effective tools.

## Introduction

Self-regulated learning (SRL) broadly refers to the phenomenon by which students can self-regulate their learning process without the direct guidance of an educator (Zimmerman and Pons, 1986; Panadero, 2017) and has been shown to have a positive impact on engagement and outcome (Cleary et al., 2006; Lee et al., 2010; Liu, 2016). There are several frameworks for SRL (Hadwin et al., 2011; Panadero, 2017), but one of the most influential is Zimmerman's cyclical phase model, which splits the processes of SRL into three phases: forethought, performance, and self-reflection (Zimmerman and Pons, 1986; Panadero, 2017). Forethought encompasses skills used to plan or set goals for a learning activity, performance encompasses skills used to complete the activity and monitor one's progress toward goals, and self-reflection encompasses skills related to evaluating one's performance and adapting it for future iterations of the activity. This model, and variations of it, have been used to study SRL in several academic contexts (Zimmerman and Pons, 1986; Magno, 2010) as well as athletic contexts including basketball (Cleary and Zimmerman, 2001; Cleary et al., 2006), dart throwing (Zimmerman and Kitsantas, 1997), and volleyball (Kitsantas and Zimmerman, 2002). These studies provided valuable insights into how SRL manifests in athletics, which sparked additional exploration of SRL outside of traditional academic contexts, such as SRL in educational games (Sabourin et al., 2013; Nietfeld et al., 2014; Nietfeld, 2017).

However, there is currently no work examining how individuals apply SRL processes in the context of esports. This is in spite of the fact that esports have evolved into a multi-billion dollar industry (Media, 2021) and demonstrated real world benefits for players (Hilvoorde and Pot, 2016; Wu et al., 2021). This popularity and perceived benefits have led to esports' recognition as an official sport (esports.net, 2021) and their adoption in educational contexts (Cho et al., 2019; Lee et al., 2020). Similar to traditional athletics, esports skill is highly dependent on a player's ability to learn and master gameplay mechanics (Donaldson, 2017; Fanfarelli, 2018), and thus, SRL processes are likely correlated with one's chances of becoming a successful player. That being said, without knowledge of how SRL manifests within esports, it is currently difficult to make informed decisions about how to support learning in the context of esports play.

There are several ways that the formal study of SRL in esports could benefit both the learning and games communities. For games, and especially for esports, knowledge of how SRL manifests within the domain could highlight opportunities to more effectively support learning through specialized training or computational tools that target SRL processes (Kuan et al., 2017; Afonso et al., 2019). Further, knowledge of where SRL processes are not being leveraged by players, or where they differ across skill levels, may highlight elements of the gameplay experience where learning is more difficult. These elements may act as obstacles to novices seeking to move into higher skill-level play. As such, identifying these pain points, and developing support systems that can address them, can help prevent feelings of frustration or inadequacy, which have been known to result in discontinuation of play (Brusso et al., 2012; Esteves et al., 2021). When implemented into the games themselves, such support may result in lower churn rates.

For learning, there are two areas where more formal understandings of SRL within esports could have substantial implications. First, there could be an opportunity to use esports to train SRL skills that could then transfer to academic contexts. Existing work has already demonstrated that esports play can improve players' emotional regulation (Wu et al., 2021), fine motor skills (Toth et al., 2021), and academic performance (Rothwell and Shaffer, 2019). As such, esports have seen increased adoption as extracurricular activities in schools (Cho et al., 2019; Lee et al., 2020). If esports players are demonstrating strong SRL skills, such as reflection or goal setting, it may be that their engagement with the games themselves is teaching these skills, and this may be another benefit warranting their inclusion in schools.

Second, esports interfaces could inspire the design of future e-learning technologies that more effectively engage students. There already exist numerous data-driven tools to improve and enhance students' learning, including Open Learner Models (OLMs) (Hooshyar et al., 2020) and Learning Analytics Dashboards (LADs) (Bodily and Verbert, 2017; Bodily et al., 2018). As esport games often include data visualization systems reminiscent of these learning technologies, a better understanding of how SRL skills manifest in relation to these could provide OLMs and LADs with valuable design insights for engaging, and even playful, systems. This, along with the previous two implications, however, requires a stronger understanding of how SRL skills and processes manifest among esports players.

The work presented here is, to the authors' knowledge, the first study to empirically examine the manifestation of processes from Self Regulated Learning in the context of esports. Specifically, we replicate the study of Kitsantas and Zimmerman (2002) that examined SRL differences between novice, non-expert, and expert volleyball players. In place of volleyball, we recruited 30 League of Legends players (10 novices, 10 non-experts, and 10 experts) and collected data regarding their self-regulatory processes and gameplay practices following the exact protocol of the original study, adapted to the new context.

Our results found that the three groups differed significantly in their execution of goal setting and planning, which are termed forethought processes by Zimmerman (Zimmerman and Pons, 1986; Panadero, 2017), but not in the other phases. We suggest that League of Legends' in-game interface features, as well as external tools that are easily accessible by players at all skill levels, may be nurturing SRL skills in the performance and self-reflection phases simply through interaction with them. By contrast, skills in the forethought phase are not prominently supported by existing tools and would instead require interaction with a team or coach to develop, making them more common among expert players. Based on these conclusions, we present three implications and avenues for future work. First, we suggest that the forethought phase presents an opportunity for the development of new computational support tools for players that could help bridge the gap between novice and expert play. Second, we suggest that esports could be used to train SRL skills that may transfer to academic contexts, and propose to explore this further in future work. Third, we suggest that data-driven systems for learning may be able to leverage design standards from esports interfaces to better engage students.

## Related Work

In this section we will frame the contribution of this work by providing an overview of previous work on Self-Regulated Learning (SRL) and SRL in games.

### Self Regulated Learning

There are several different theories of SRL, which, broadly, all encompass the processes and skills related to analyzing tasks, setting goals, developing strategies to reach those goals, monitoring progress toward those goals, and reviewing performance and outcomes (Puustinen and Pulkkinen, 2001; Boekaerts and Corno, 2005; Künsting et al., 2011; Winne, 2011; Panadero, 2017). The models vary, however, in how they conceptualize each aspect of SRL and what skills they emphasize (Panadero, 2017). Perhaps the most influential of SRL models, however, is Zimmerman's Cyclical Phase Model, which has influenced much of the SRL work and models that have come after (Zimmerman, 2000; Puustinen and Pulkkinen, 2001; Zimmerman and Campillo, 2003; Panadero, 2017). The Cyclical Phase Model is often used in the literature to study SRL in various academic contexts (Barnard-Brak et al., 2010; Lee et al., 2010; Malmberg et al., 2017; Min and Foon, 2019) and has been built upon by more recent SRL models (Hadwin et al., 2011; Panadero, 2017).

**Figure 1:**
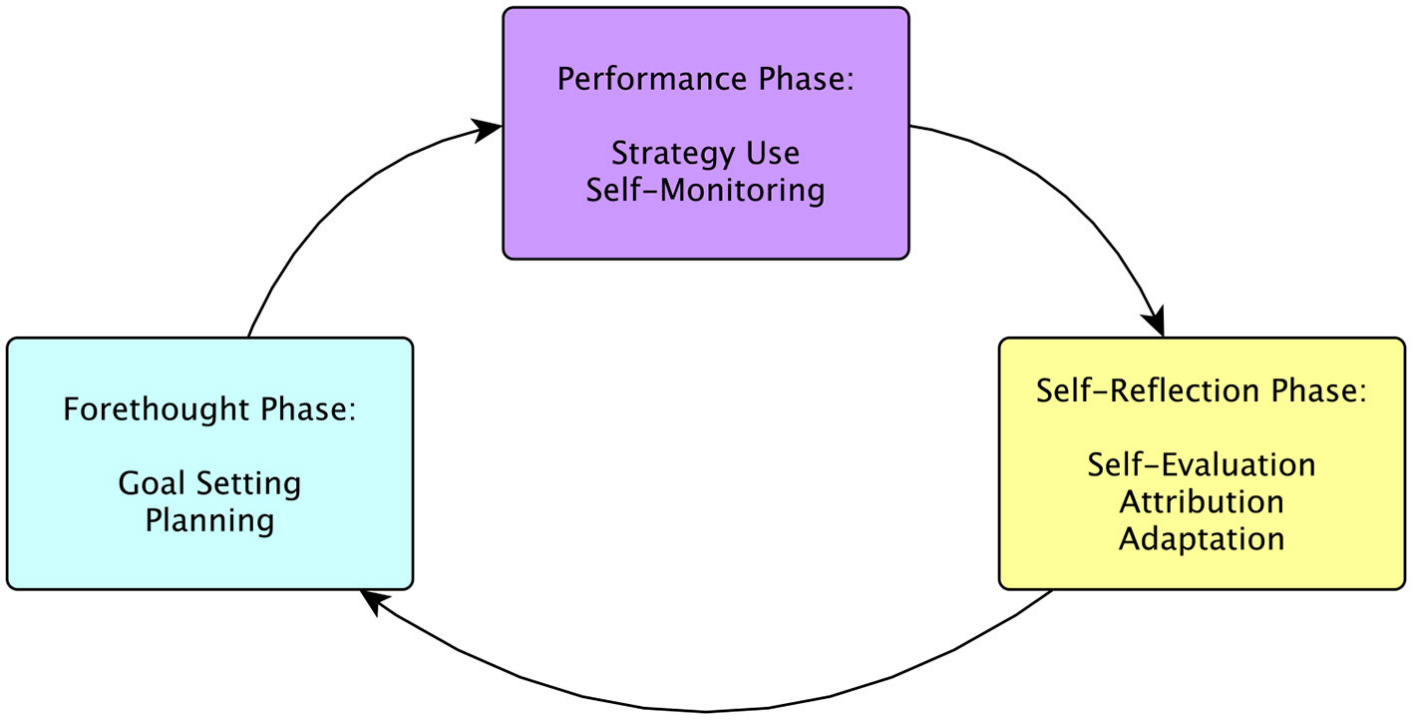
An overview of the cyclical phase model of self-regulated learning highlighting the specific processes examined in this work and which phase they belong to.

Building on Zimmerman's earlier models of SRL (Zimmerman and Pons, 1986; Zimmerman and Kitsantas, 1997; Magno, 2010), The Cyclical Phase Model organizes SRL processes into three phases: forethought, performance, and self-reflection (Zimmerman, 2000; Zimmerman and Campillo, 2003). An overview of this model can be seen in Figure 1. The forethought phase includes processes such as analyzing the task, setting goals, and planning how to reach them. The performance phase encompasses execution of the task and progress monitoring along with strategies to maintain engagement and motivation. The self-reflection phase encompasses the processes by which the learner assesses how they performed the task (Zimmerman, 2000; Zimmerman and Campillo, 2003; Panadero, 2017).

Zimmerman and his research partners also demonstrated the relevance of the Cyclical Phase Model beyond academics, through several studies that used it to examine SRL in athletic contexts (Zimmerman and Kitsantas, 2005; Zimmerman, 2006). In a 2001 study, Cleary and Zimmerman found that expert basketball players set more specific goals and technique-oriented strategies during the forethought phase and more often attributed failure to faulty technique during the self-reflective phase than non-expert or novice players (Cleary and Zimmerman, 2001). Building on this, in 2006, Cleary and Zimmerman used the Cyclical Phase Model in a study that examined the impact of the additive effects of self regulation training in forethought, performance, and self reflection processes on basketball free-throws (Cleary et al., 2006). They found that those who practiced all three phases of SRL had a significantly better shooting performance than those who only practiced one phase or none, indicating that SRL had a significant impact on overall performance (Cleary et al., 2006).

Most relevant to this work, however, is a 2002 study by Kitsantas and Zimmerman (2002) that used the Cyclical Phase Model to study differences in SRL between expert, non-expert, and novice volleyball players. They conducted a micro-analytic study in which players were asked questions about their general practice techniques for learning and mastering overhand serves. They were then asked to perform before the researchers and answer additional questions about how they felt they did and why they may have failed (Kitsantas and Zimmerman, 2002). The results found that experts set better goals and had better planning during the forethought phase, better strategy use and self-monitoring during the performance phase, and better evaluations, attributions and adaptations during the self-reflection phase than either non-experts or novices (Kitsantas and Zimmerman, 2002).

Together, this literature paints a clear picture of the significant role that SRL plays in athletics, which can be used to better understand how to help athletes gain expertise (Zimmerman and Kitsantas, 2005; Zimmerman, 2006). However, with the rise of esports comes the question of whether or not these findings are applicable to the new domain. Unfortunately, this remains an open question, as much of the work on SRL in the context of digital games focuses almost exclusively on educational games (Nietfeld, 2017).

### Self Regulated Learning in Games

In the context of digital games, SRL is notably under-studied, and much of the existing work focuses almost entirely on educational games (Nietfeld et al., 2014; Nietfeld, 2017). For example, Sabourin et al. (2013) generated SRL scores for students who played the educational game *Crystal Island* (Rowe et al., 2009) based on their responses to a reflective prompt. They found that SRL scores were significantly predictive of post-test learning gains and that high-SRL students appeared to make more use of the in-game curricular resources than low-SRL students and reported more immersion, interest, and enjoyment (Sabourin et al., 2013).

Reflective, or self-explanation prompts, are, in fact, one of the most common ways that self-regulated learning has been implemented in educational games, and some of the literature has studied the impact of this design (Nietfeld, 2017). For example, ONeil et al. (2014) added a self-explanation prompt, which encouraged self-reflection processes, to an educational math game and found that students who responded to the prompts tended to have higher mean post-test scores than those who did not. Similarly, Fiorella and Mayer (2012) found that adding prompts to a game that taught electrical circuits significantly increased student performance.

Another area of interest for SRL in games is goals (Nietfeld, 2017). This is unsurprising, given that gameplay is often driven by the pursuit of goals (Lankoski, 2011). Several studies have examined the impact of different kinds of goals on performance in game-based learning. For example, Künsting et al. (2011) examined the impact of type and specificity of goals in a game-based learning environment that taught buoyancy concepts. Their results found that non-specific problem-solving goals yielded substantially more frequent strategy use from learners, but that this was not the case when the goals were learning goals (Künsting et al., 2011). Feng and Chen (2014) examined a similar question, but in the context of educational game design through Scratch (Resnick et al., 2009). Their results demonstrated that students with non-specific goals outperformed those with more specific goals and that students with structuring scaffolds demonstrated worse SRL (Feng and Chen, 2014).

While all of this work demonstrates the role that SRL can and does play in games, it focuses entirely on educational games and, in most cases, on the impact SRL has on players' learning of the educational content (Künsting et al., 2011; Fiorella and Mayer, 2012; Feng and Chen, 2014; ONeil et al., 2014). In contrast, there is currently little work that examines the role that SRL plays in learning the skills and mechanics involved in playing a game. Brusso et al. (2012) provide one of the only examples of work that examines SRL skills in relation to gameplay performance itself. In their study, they investigated the impact of unrealistic performance goals on player performance in a first-person-shooter game (Brusso et al., 2012). They found that those whose performance fell short of their goal would perform significantly worse in subsequent levels than those whose performance more closely matched their goal. Further, they found that this was significantly more common for those players with high video-game self-efficacy (Brusso et al., 2012). This demonstrates that SRL plays a role in games beyond education. Specifically, SRL skills, such as goal setting, can have an impact on gameplay performance. In the context of esports, knowledge of the role and impact of SRL would be invaluable to helping players more effectively learn and gain expertise, which is often cited as the primary motivation for the development of computational support tools for esports (Wallner and Kriglstein, 2016; Kuan et al., 2017; Afonso et al., 2019).

## Methods

We chose Kitsantas and Zimmerman (2002)'s study as it provided a foundational overview of how SRL skills relate to expertise in athletics, which we felt was transferable to the esports context. Additionally, we found the protocol to be easily translatable to League of Legends, a digital, online game. The study was carried out from May to July 2021 and COVID19 required the study to be conducted remotely over Zoom. In this section we will outline the steps we took to recreate this study.

### League of Legends

**Figure 2:**
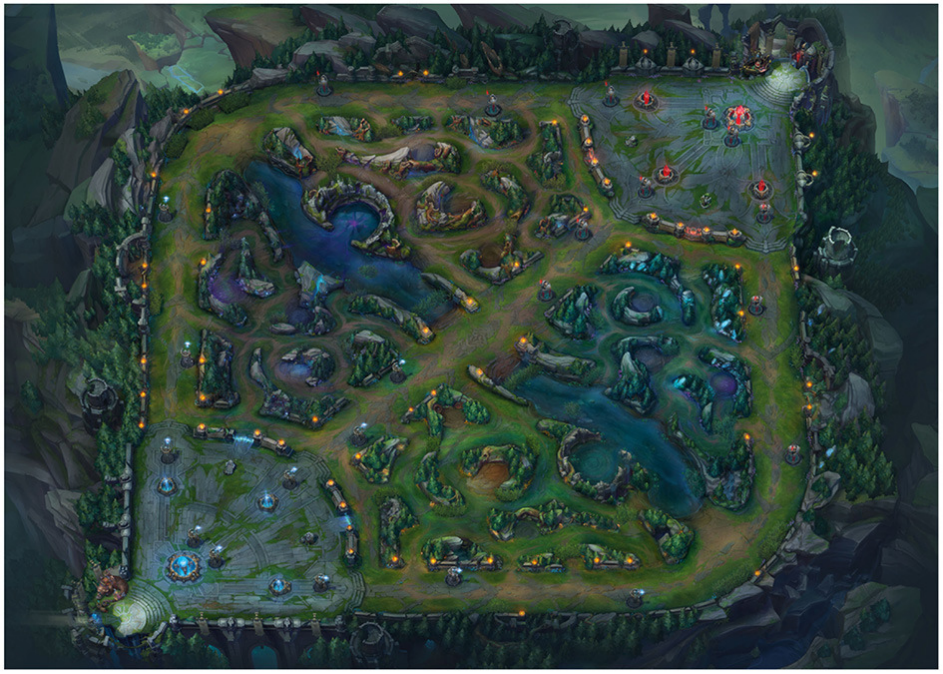
The league of legends game map. On the bottom left is the base for the green team, on the top right is the base for the red team. These are linked by three lanes, and the areas between the lanes are the jungles. The lanes contain towers that fire at enemy players and must be destroyed to advance toward the enemy base. Image reproduced with permission from Riot Games Inc. This image is copyrighted to Riot Games Inc.

League of Legends is an esport game developed by Riot games and belonging to the Multiplayer Online Battle Arena (MOBA) genre. The game is played by two teams of five on a square map (see Figure 2) where each team has a base in either the lower left (for the green team) or upper right (for the red team) corner of the map. The bases house a crystal called a “Nexus” and the goal for each team is to reach and destroy the opposing team's nexus. The rest of the map consists of three lanes which extend from base to base and are referred to as top (for the one that follows the left and top edges of the square map), middle (for the one that cuts diagonally across the center of the square), and bottom (for the one that follows the bottom and right edges of the square map). There are also forested areas between the lanes, referred to as the “jungle.”

A League of Legends team typically has players play on one of five designated roles: top (usually a high defense character, starts in the top lane), mid (usually a powerful attacker, starts in the mid lane), jungler (usually a character with high mobility and skills like health regeneration that help it survive, moves around the jungles), adc (another powerful attacker, starts in the bottom lane), and support (usually has healing or shielding abilities, helps the adc in the bottom lane). The three lanes each house six towers (three for each team) that fire lasers at opposing entities and must be destroyed in order to reach the enemy base. The jungle, by contrast, is home to various monsters that can be killed for gold or experience points.

In order to win a match of League of Legends, players must gain experience to level up their characters, gold to buy items to make their characters stronger, and win battles against enemy players in order to destroy the opposing towers and advance across the map. We chose League of Legends for the study due to its immense popularity as an esport game (leaguefeed.net, 2021). Additionally, because it is part of the extremely popular MOBA genre of esports, which also includes titles such as DOTA2 and Heroes of the Storm, we believed the results of this study would be indicative of general trends across multiple games.

### Last Hitting

We chose last hitting as the skill to focus on in this study, whereas the original study focused on overhand serves. In League of Legends, there are small non-player character (NPC) entities called minions or “creeps,” which march down the lanes from the two bases and attack nearby enemy minions, players, or towers and grant gold and experience to players when killed. More gold and experience are awarded to the player who deals the finishing blow, which is advantageous to players as the experience allows them to level up and the gold can be used to buy equipment, both of which make them stronger. The act of intentionally dealing finishing blows is referred to as “last hitting” and is a strategic maneuver that involves carefully timing one's attacks to ensure that the finishing blow to an enemy minion can be dealt by the player rather than their own minions or tower. The number of minions a player has last hit is referred to as their creep score (CS). Last hitting is widely considered to be relatively easy to understand, but difficult to master from a technical standpoint, which makes it similar, conceptually, to the overhand serves that were the focus of Kitsantas and Zimmerman's volleyball study (Kitsantas and Zimmerman, 2002).

### Recruitment

30 League of Legends players were recruited to participate: 10 experts, 10 non-experts, and 10 novices, following the participant breakdown of the original study. In the context of League of Legends, the line between expert, non-expert, and novice is not as clear as it is in traditional sports contexts. Further, complete beginners would lack the technical knowledge (i.e., how to navigate or attack in-game) to complete the study's steps. Thus, we worked with a collegiate League of Legends coach to define the three skill levels based on criteria appropriate to the esports context. These were as follows:

- Expert referred to someone who currently played (or had played in the past) on an established, competitive team and participated (or had participated in the past) in formal competitions.
- Non-Expert referred to someone who currently played (or had played in the past) with one or multiple informal teams (i.e., a team comprised of a group of friends) in a recreational manner, who may have played ranked games but did not participate in formal competitions.
- Novice referred to someone who only played on pick-up teams (i.e., solo-queue) and did not play competitively at any point.

Skill level was self-reported by prospective participants when filling out an online recruitment form. The 10 experts were recruited from collegiate League of Legends teams at several North American universities. The non-experts and novices were recruited through convenience sampling and social media ads. All participants received a 30$ Amazon gift card as compensation.

27 participants identified as male, 2 as female, and 1 as non-binary. Age ranged from 18 to 39 and the average age was 22. Race information was not collected. The average age for experts was 20.1, for non-experts was 21.9, and for novices was 24.5. On average, expert players had 5.75 years of experience, non-experts had 4.8, and novices had 4.9. Novices had a slightly higher average due to several players who had been playing for a long time but never beyond the novice level. Across the entire sample, 2 players played jungle (both experts), 8 played top lane (two experts, three non-experts, and three novices), 5 played mid lane (three non-experts and two novices), 9 played adc (five experts, one non-expert, and three novices), 5 played support (one expert, two non-experts, and two novices), and one (a non-expert) played fill (all positions).

To assess their general knowledge of last hitting, participants were asked to describe a last hit “as if they were explaining it to someone who did not know what it was.” Responses were written down verbatim by the lead researcher. These were scored according to a rubric developed in collaboration with a League of Legends coach. Participants' responses were awarded a point for each of the following they mentioned:

- Timing (when to strike the minion)
- Wave management (pushing, pulling, or freezing the wave)
- Trade patterns/opponent presence (knowing how to last hit around an enemy)
- Champion/role differences (different characters and roles CS differently).

As there were four elements, participants could score up to four points, however, no player scored perfectly. The mean of experts' knowledge of last hitting was 1.8, the mean of non-experts' knowledge was 1.3, and the mean for novices' knowledge was 1.4. The low means are likely the result of the lack of a definitive definition of the skill within the gameplay community, with many players knowing what it is and how to perform it but struggling to articulate it in words. The low means are not of concern to this study, as all players were shown the same instructional video on how to perform a last hit after giving their description.

### Procedure

Following the collection of demographic data and knowledge of last hitting, in line with the protocol used by Kitsantas and Zimmerman (2002), all participants were shown the same video about how to execute a last hit. They were then asked a set of questions regarding their self-efficacy, perceived instrumentality of last hitting, intrinsic interest in last hitting, goal setting, and planning (measures described below).

Following these questions, participants were instructed to open the League of Legends practice tool, where they could create a custom, solo game with no other players and practice last hitting for 10 min. Participants were instructed to share their screen at this point so the attending researcher could observe. Participants used their own accounts and were instructed to select any character that they were comfortable last hitting with, to ensure that familiarity with the character's skills would not confound the results. They were instructed to buy their usual starting equipment when the game loaded and proceed to the middle lane. They were also instructed not to leave the lane to explore the jungle or buy more equipment. This was to ensure that all participants spent the same amount of time last hitting in the lane. Following the practice session, participants were asked about their strategy use, self-monitoring, self-evaluation, and self-satisfaction during the session (measures described below).

Participants were then tested for last hitting skill, and asked to create a second game in the practice tool with the same arrangement. This time, however, they would only last hit until they missed a last hit. All participants did miss a last hit. At this point they were asked about their attributions, adaptation processes, and self-efficacy perceptions (measures described below). Participants were then debriefed and thanked by the researcher, who also answered any questions they had about the study. Participants received their payment via email after completion of the session. The protocol was carried out by one researcher and was reviewed and approved by the university's institutional review board.

### Measures

#### Last Hitting Skill

League of Legends tracks how many last hits a player has achieved in a user-interface (UI) element in the upper right corner of the screen. Last hitting skill was evaluated based on this number at the point at which the player missed the last hit during the second custom game.

#### Measures of Self Motivation

The questions for the measures of self-motivation were adapted directly from those used by Kitsantas and Zimmerman (2002). All participants were asked the following questions to measure the respective factors:

- “On a scale from 0 to 100 with 10 being Not Sure, 40 being Somewhat Sure, 70 being Pretty Sure, and 100 being Very Sure, how sure are you that you are able to last hit every creep in a given wave?” (Self-Efficacy). This was asked once before practice and again after missing a last hit during the second custom game.
- “How interesting is last hitting to you on a scale from 0 to 100 with 10 being Not Interested, 40 being Somewhat Interested, 70 being Pretty Interested, and 100 being Very Interested” (Intrinsic Interest). This was asked once before practice.
- “How important is last hitting skill in attaining your future goals on a scale from 0 to 100 with 10 being Not Important, 40 being Somewhat Important, 70 being Pretty Important, and 100 being Very Important” (Perceived Instrumentality). This was asked once before practice.
- “On a scale from 0 to 100 with 10 being Not Satisfied, 40 being Somewhat Satisfied, 70 being Pretty Satisfied, and 100 being Very Satisfied, how satisfied are you with your performance during this practice session?” (Self-Satisfaction). This was asked once after practice.

#### SRL: Forethought Phase

**Goal Setting:** Before practice, all participants were asked “Do you set any specific goals for your sessions when practicing last hitting and if yes, what are they?” The researcher recorded the answer verbatim. The goals were then coded independently by two researchers into one of the following categories: outcome goals, technique of process goals, other, and no goals, the same scale used by Kitsantas and Zimmerman (2002). For the context of League of Legends, the categories were considered as follows:

- “Outcome goals” referred to statements related to getting a certain number of last hits or amount of gold.
- “Process goals” referred to statements related to managing opponent presence or number and positioning of creeps in the lane.
- “Other” referred to any statements that did not discuss either of the above.

These definitions were developed and agreed upon by two researchers with extensive League of Legends experience. Cohen's kappa (Cohen, 1960) was used to check for agreement and resulted in a score of .9, indicating very strong agreement (Landis and Koch, 1977).

**Planning:** Also before practice, participants were asked “Do you have a regular routine that you follow when you practice on your own?” The responses were again recorded verbatim and coded by two researchers into one of the following categories: completely structured routine, partially structured routine, or unstructured routine, the same scale used by Kitsantas and Zimmerman (2002). For the context of League of Legends, these were defined as follows:

- A “completely structured routine” referred to discussions of regular practice using the practice tools or regularly playing warm up games in less competitive game modes.
- A “partially structured routine” referred to discussions of staying in practice by just playing regularly or irregular practice sessions.
- An “unstructured routine” referred to discussions of not practicing.

These definitions were developed and agreed upon by the same two researchers with extensive League of Legends experience. There were no disagreements in the code applications resulting in a kappa value of 1, indicating perfect agreement (Landis and Koch, 1977).

#### SRL: Performance Phase

**Strategy Use:** Two questions were asked regarding strategy use, echoing Kitsantas and Zimmerman's protocol (Kitsantas and Zimmerman, 2002). These were:

- “What do you need to do to accomplish your goals?” (Asked before practice)
- “What do you need to do to successfully execute the last hit next time?” (Asked after missing a last hit during the second custom game).

These were again recorded verbatim and coded by two researchers into one of the following categories: specific technique, visualization strategies, concentration strategies, both, and practice/no strategies, the scale used by Kitsantas and Zimmerman (2002). For the context of League of Legends these were defined as follows:

- “Specific technique” referred to discussions such as getting the timing right, using the right skill, or targeting the right minion.
- “Visualization strategies” referred to any discussion of visualizing or imagining oneself doing it correctly.
- “Concentration strategies” referred to any discussion of focusing or concentrating either in general or on a specific aspect of gameplay.
- “Technique and concentration” referred to responses that included both.
- “Practice/no strategy” referred to answers that just discussed practicing or did not discuss any strategy.

These definitions were developed and agreed upon by the same two researchers. Cohen's kappa resulted in a score of .91 for the first question and .83 for the second, both indicating very strong agreement (Landis and Koch, 1977).

**Self-Monitoring:** After the practice session, all participants were asked “How did you monitor your performance and progress during the practice session?” These were again recorded verbatim and coded by two researchers into one of the following categories: creep score alone (corresponding to Kitsantas and Zimmerman's ‘service outcome points alone’), use of technique or form and its outcomes, do not know, or other, the scale used by Kitsantas and Zimmerman (2002). For the context of League of Legends these were defined as follows:

- “Creep score alone” referred to discussions of tracking the number of last hits achieved, either in one's head or using the UI's CS score board.
- “Use of technique or form and its outcomes” referred to discussions of technical execution of the skill, such as making sure the minions were in the right spot or managing their numbers.
- “Do not know” referred to statements indicating that they did not monitor their performance or were not sure if they did.
- “Other” referred to any self monitoring strategy that did not correspond with the above.

These definitions were developed and agreed upon by the same two researchers. There were no disagreements in the code applications resulting in a kappa value of 1, indicating perfect agreement (Landis and Koch, 1977).

#### SRL: Self-Reflection Phase

**Self-Evaluation:** Also after the practice session, participants were asked “Did you evaluate your performance during the practice session? If so, how?” These were again recorded verbatim and coded by two researchers into one of the following categories:

- Self-evaluator (if they responded yes and gave a reasonable example of self-evaluation)
- Non-self-evaluator (if they responded no or failed to give a reasonable example of self-evaluation).

These are exactly the categories used by Kitsantas and Zimmerman (2002) and did not need to be adjusted to the context of League of Legends due to the general definitions. There were no disagreements in the code applications resulting in a kappa value of 1, indicating perfect agreement (Landis and Koch, 1977).

**Attributions:** After missing a last hit, participants were asked “Why do you think you missed the last hit?” These were again recorded verbatim and coded by two researchers into one of the following categories: form or technique, power, ability, practice, concentration, and do not know, the scale used by Kitsantas and Zimmerman (2002). For the context of League of Legends, these were defined as follows:

- “Form or technique” referred to discussion of strategic failures such as wave or health management or player positioning.
- “Power” referred to discussion of physical failures such as reaction time or mis-clicks.
- “Ability” referred to discussion of one's gameplay skill.
- “Practice” referred to discussions of practice (i.e., needing more).
- “Concentration” referred to discussions of focus.

These definitions were developed and agreed upon by the same two researchers. Cohen's kappa resulted in a score of 0.78, indicating strong agreement (Landis and Koch, 1977).

**Adaptation:** After missing a last hit, all participants were asked the following three questions, answered with either a “yes” or “no,” following Kitsantas and Zimmerman's protocol (Kitsantas and Zimmerman, 2002):

- “After missing last hits, do you think about why you missed?”
- “When you miss a last hit, do you change anything during your next attempt?”
- “If you repeatedly miss last hits, do you ask your coach or teammates to give you feedback or advice?”

## Results

Shapiro-Wilk tests were used to check for normal distributions of the numerical self-motivation data. Test results indicated that the data was not normally distributed, and thus non-parametric Mann-Whitney tests and Kruskal-Wallis tests were used for these data. Chi-square tests were used to assess differences for categorical data.

Between knowledge of last hitting, years of experience, and age, only years of experience was normally distributed. According to two-tailed *t*-tests used for years of experience, and Mann-Whitney tests used for knowledge and age, there were no significant differences between groups.

### Last Hitting Skill

Last hitting skill was determined using the creep score, or number of creeps last hit, each player earned during the second custom game (when they were asked to last hit until they missed one). The means and standard deviations for each group are shown in Table 1. Kruskall-Wallis results indicate that the differences between all three groups are not statistically significant (*p* > 0.05).

**Table 1:** The means and standard deviations for creep score for each group.

**Group**	**Mean**	**STDEV**	**Median**
Experts	17	12.9	11.5
Non-experts	15.1	19.1	9
Novices	8.4	12	5

### Measures of Self-Motivation

Due to the non-normal distribution of the data, Kruskall-Wallis tests with Bonferroni corrections were used to check for significant differences for each variable. The means and standard deviations are shown in Table 2. Kruskall-Wallis results indicated that the differences between groups were not significant (*P* > 0.05) for all measures except for the Self-Efficacy (Before Practice) measure (*H* = 8.35, *P* = 0.01, Degrees of Freedom = 2). Mann-Whitney pair-wise test results with Bonferroni corrections indicate that experts had significantly higher self-efficacy at this point than novices (*U* = 79, *P* = 0.01). Non-experts did not differ significantly from novices or experts at this point (*P* > 0.016). There were no significant differences between groups at the second self-efficacy measurement due to the changes in mean and standard deviation. Mann-Whitney results indicate that these changes from before to after were also not significant.

**Table 2:** The means and standard deviations for the five measures of self-motivation.

**Variable**	**Experts**	**Non-experts**	**Novices**
**Self-efficacy (Before practice)**			
Mean	79	58	58
St. dev	14.5	21	15.5
Median	70	70	70
**Self-efficacy (After missing)**			
Mean	76	55	55
St. dev	19	25.5	25.5
Median	70	70	55
**Intrinsic interest**			
Mean	64	43	70
St. dev	31	26.3	24.5
Median	70	40	70
**Perceived instrumentality**			
Mean	91	79	82
St. dev	14.5	20.2	25.3
Median	100	70	100
**Self-satisfaction**			
Mean	73	73	58
St. dev	17	17	25.3
Median	70	70	70

### Self-Regulated Learning Processes

In the following sub-sections we discuss the results regarding how players at different skill levels engaged SRL processes across the three phases of Zimmerman's model.

#### Forethought Phase

**Goal Setting:** There were significant differences in goal setting among the three expertise groups [χ^2^_(6)_ = 13.1, *P* = 0.04]. The counts for each goal type for each skill level can be seen in Table 3. Cramer's V was calculated to determine effect size and the result (*w* = 0.46) indicates a medium to large effect size.

**Table 3:** An overview of how different types of goals were set across the three skill levels.

**Forethought: Goal setting**	**Experts**	**Non-experts**	**Novices**
Outcome goals	5	6	0
Process goals	3	2	7
Other goals	0	2	1
No goals	2	0	2

**Planning:** There were significant differences in planning among the three expertise groups [χ^2^_(4)_ = 14, *P* = 0.007]. The counts for each goal type for each skill level can be seen in Table 4. Cramer's V was calculated to determine effect size and the result (*w* = 0.48) indicates a medium to large effect size.

**Table 4:** An overview of how different routines were used across the three skill levels.

**Forethought: Planning**	**Experts**	**Non-experts**	**Novices**
Completely structured	5	1	0
Partially structured	4	5	2
Unstructured	1	4	8

#### Performance Phase

**Strategy Use:** There were no significant differences for strategy use before practice [χ^2^_(8)_ = 6.94, *P* > 0.05] or after missing last hits [χ^2^_(4)_ = 4.26, *P* > 0.05]. The counts for each strategy type for each skill level can be seen in Table 5. For the second question, asked after missing last hits, “Visualization Strategies” and “Practice/No Strategy” were never applied to the participants' statements by the two researchers.

**Table 5:** An overview of how different strategies were used across the three skill levels at both question times.

**Performance: Strategy use**	**Experts**	**Non-experts**	**Novices**
**Before practice**			
Specific techniques	2	4	4
Visualization	2	0	0
Concentration	3	3	1
Technique and concentration	1	1	1
Practice/None	2	2	4
**After missing**			
Specific techniques	5	6	6
Concentration	1	3	3
Technique and concentration	4	1	1

**Self-Monitoring:** There were no significant differences between the groups for self-monitoring [χ^2^_(4)_ = 5.97, *P* > 0.05]. The counts for each technique for each skill level can be seen in Table 6. “Do Not Know” was never applied to the statements by the two researchers.

**Table 6:** An overview of how different self-monitoring techniques were used across the three skill levels.

**Performance: Self-monitoring**	**Experts**	**Non-experts**	**Novices**
Points	9	5	6
Technique	1	5	3
Other	0	0	1

#### Self-Reflection Phase

**Self-Evaluation:** There were no significant differences between the groups for self-evaluation [χ^2^_(2)_ = 2.2, *P* > 0.05]. The counts for self-evaluation for each skill level can be seen in Table 7.

**Table 7:** An overview of how self-evaluation occurred across the three skill levels.

**Reflection: Self-evaluation**	**Experts**	**Non-Experts**	**Novices**
Yes	9	8	10
No	1	2	0

**Attributions:** There were no significant differences between the groups for attribution [χ^2^_(4)_ = 0.6, *P* > 0.05]. “Ability,” “Practice,” and “Do Not Know” were never applied to the attribution statements by the two researchers. The counts for the remaining attribution types across the skill levels can be seen in Table 8.

**Table 8:** An overview of attribution types across the three skill levels.

**Reflection: Attributions**	**Experts**	**Non-experts**	**Novices**
Form and technique	5	5	5
Power	3	3	4
Concentration	2	2	1

**Adaptation:** The responses for the three adaptation questions can be seen in Table 9. Chi square tests indicated no significant differences between groups (all *P* > 0.05).

**Table 9:** The number of people in each group who said yes and no for each of the adaptation questions.

**Reflection: Adaptation**	**Experts**	**Non-experts**	**Novices**
Do you think about it?			
Yes	6	5	5
No	4	5	5
Do you change anything?			
Yes	4	7	7
No	6	3	3
Do you ask for help?			
Yes	3	4	4
No	7	6	6

## Discussion

Our findings suggest that the only significant differences in SRL processes between League of Legends skill levels exist in the forethought phase. In this section, we discuss possible reasons for these findings, and their implications on future work.

### Differences Between Expertise Levels and Contexts

We observed significant differences in how players engaged SRL processes in the forethought phase. Specifically, novices discussed process goals more than non-experts and experts. This is in line with previous work that found that players seemed to shift from process to outcome goals as they obtained more skill (Zimmerman and Kitsantas, 1997). We also saw, however, that there was one more non-expert than expert who mentioned outcome goals. While we can make no real claims based on a one-participant difference, it is possible that, in the domain of esports, there may be a shift back toward process goals at the highest skill levels. This is somewhat supported by some of the statements made by expert players who discussed process goals, for example “I'm not that focused on last hitting to get the minions because I find that somewhat easy, like it comes second nature to me now, there's other stuff I take into more account when I play and try to secure my farm. So like uh wave management, mainly, that's more important to me than last hitting to secure minions, and obviously just like not screwing up the lane and dying randomly” (Participant 16, Expert). This may be because the desired outcome for last hitting is generally understood to be about 10 creeps per minute (for a total of 100 at 10 min). It may be that high-level players understand this as their desired outcome and revert to focusing on process in order to identify execution errors that may hinder it. Another quote from an expert player that suggests this is “[I will] see if I can get all of the CS when the wave is sitting in the middle, when I'm pushing, freezing, when I'm under tower. There's so many scenarios for where the minion wave is at and I want to make sure I can adjust and reach goals in every situation.” (Participant 4, expert).

For planning, we observed that more advanced players had significantly more structured practice routines than novice players. This is likely the result of novice players being less interested in competitive play, and therefore less motivated to pursue structured practice, instead choosing to play games for leisure, as articulated by Participant 22 (novice): “No, usually I just jump right into a game and go from there.” Further, advanced players are more likely to play in formal contexts, on teams or with coaches, making it easier for them to access structured practice routines, or those who can design them, than novices, who are often playing on their own. These findings are consistent with those discussed by Kitsantas and Zimmerman (2002), suggesting that this is an area where esports and traditional sports share common ground. In other words, our results indicate that novice League of Legends players, like novice volleyball players, are more often engaged in casual play than structured training.

**Figure 3:**
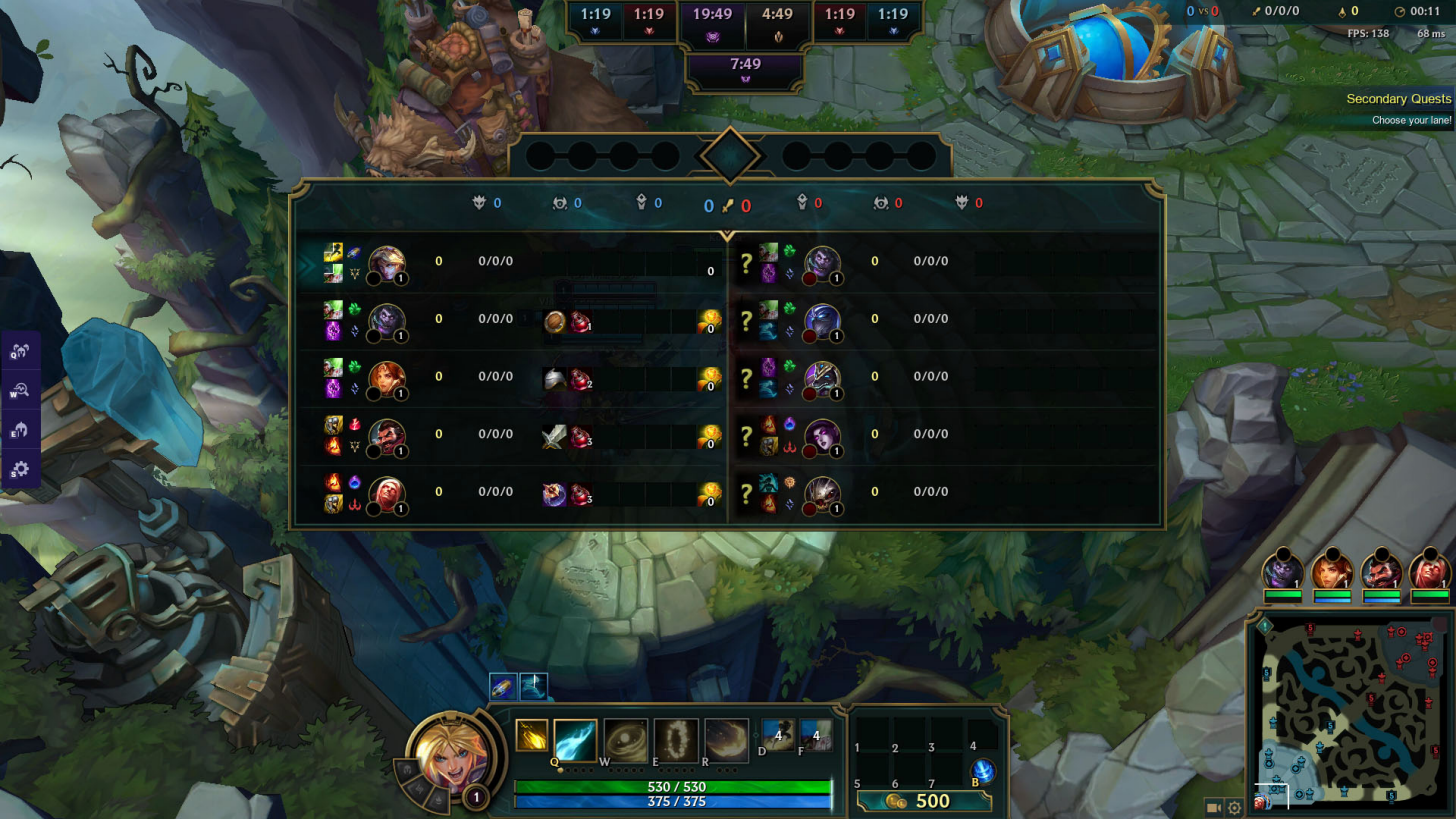
The League of Legends in-game UI presents information about player performance including kill counts, gold, experience earned, and creeps killed while playing the game. Image reproduced with permission from Riot Games Inc. This image is copyrighted to Riot Games Inc.

Interesting, however, is that there were no significant differences in SRL processes for any other phase of SRL or for the measures of self-motivation, which are in sharp contrast to the findings of Kitsantas and Zimmerman (2002), which indicated significant differences across all phases. A likely explanation for the lack of differences in the performance and self-reflection phases may be found in the design of League of Legends itself. During play, the game tracks all participating players' progress information including gold amounts, level, enemy players killed, and, of course, creeps killed. This information is visible to any player in the game either in small menus on the boarder of the screen or through a dashboard that can be accessed with the press of a button, see Figure 3. Participant responses to the self-monitoring and self-evaluation questions indicated that they were making use of these interface elements to monitor their progress during play, and that they do so on a regular basis. For example: “biggest thing is just looking at my CS vs. time elapsed” (Participant 19, non-expert) and “Mainly I just check the scoreboard, check my CS and stuff” (Participant 29, novice). Thus, it is possible that the design of the game itself is encouraging players to engage in self-monitoring practices whenever they play.

**Figure 4:**
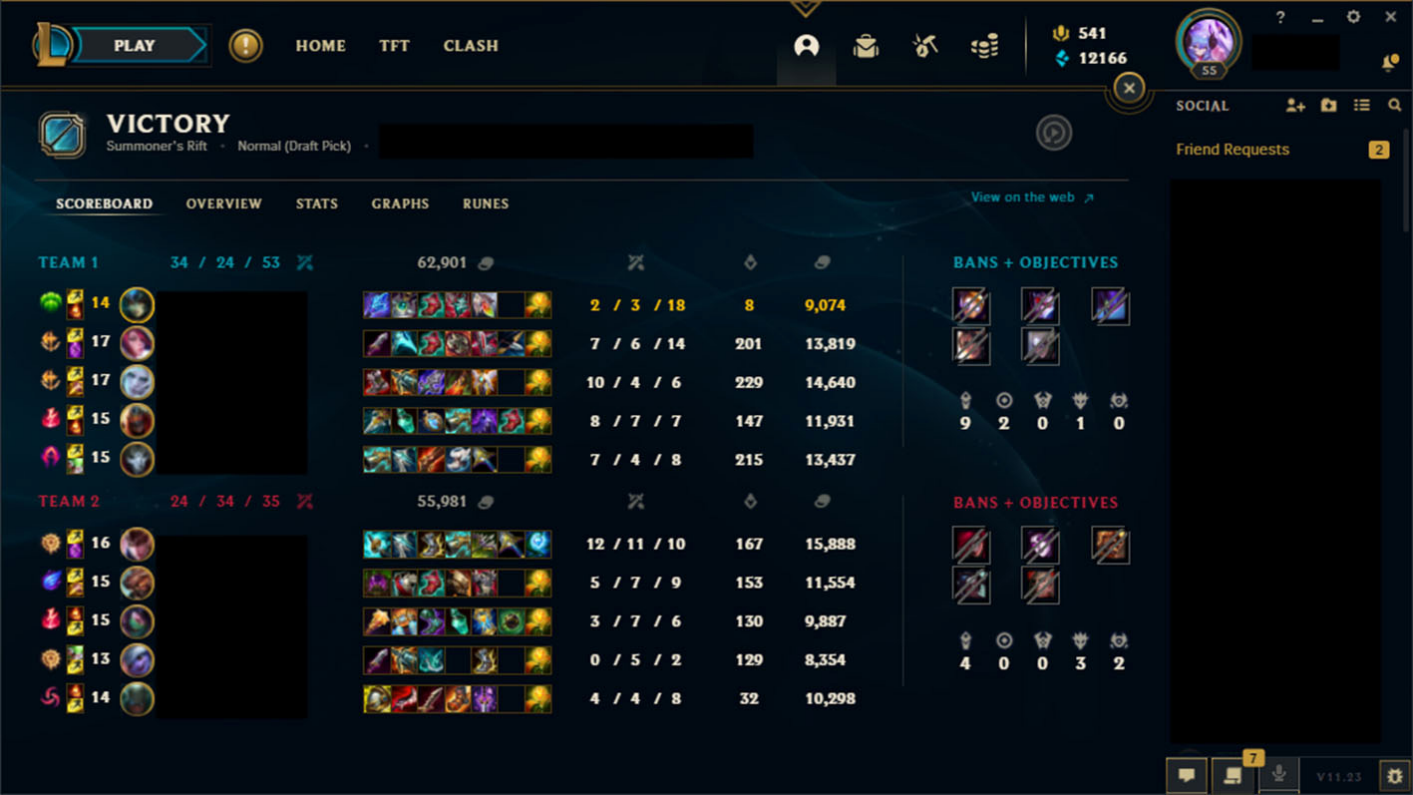
The League of Legends post-game UI presents information regarding how each player performed during the game. Image reproduced with permission from Riot Games Inc. This image is copyrighted to Riot Games Inc.

**Figure 5:**
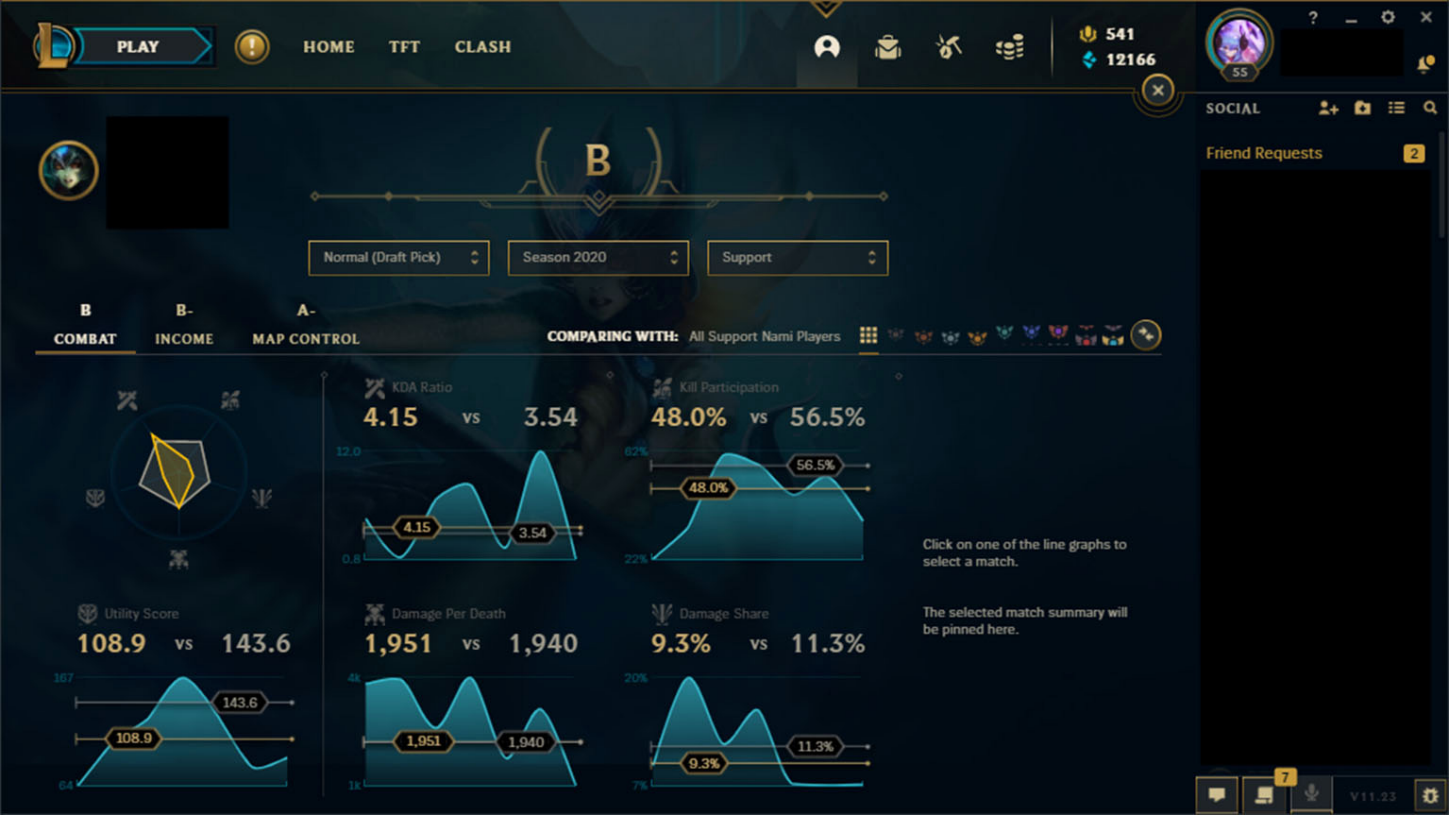
The game client stores and aggregates statistical data to present players with overviews of their gameplay over time. Image reproduced with permission from Riot Games Inc. This image is copyrighted to Riot Games Inc.

A similar situation may be the reason for the lack of significant differences in the self-reflection phase. When a match of League of Legends ends, all players are brought to a post-game screen that depicts how much each player in the game contributed and offers an assessment of their performance, see Figure 4. Additionally, the game client features a statistics interface that stores players' performance data over time and presents it to the player in aggregate graphs that depict how the player performs in comparison to other players, see Figure 5. There also exist a number of third-party tools that present players with similar information, outside of the game client (Blitz.gg, 2021; Mobalytics, 2021; SENPAI.gg, 2021). While participant responses did not mention these screens or tools in the context of the study, it is likely that their presence encourages players to reflect on their performance, especially the post game screen, which is automatically shown to all players upon completion of a match. Players who are particularly motivated to improve at the game likely spend a fair amount of time interacting with these screens in order to extract actionable insights. In other words, these screens likely encourage players to engage in self-reflection processes.

These observations resonate with existing theoretical discussions on the role of visualized data within the player experience (Medler, 2011; Medler and Magerko, 2011; Bowman et al., 2012; Hazzard, 2014) and personal informatics and quantified self in the context of games (Kou and Gui, 2018; Rapp, 2018). Specifically, previous work has discussed how game data visualization, specifically player dossiers, which present players with data on their gameplay performance over time, motivates continuous play and facilitates improvement (Medler, 2011; Bowman et al., 2012). The results of this study suggest that the improvement that comes about as a result of interaction with this visualized data may be because the visualizations encourage the execution of self-regulated learning processes. This suggests further opportunities to support players seeking to improve at gameplay through the development of visualizations of their gameplay data.

In summary, players are engaging SRL processes in the performance and self-reflection phases simply through interaction with the game's interface. Because all players at all skill levels interact with the same interface, there are few significant differences. With this in mind, the significant differences in planning and goal setting are likely the result of the game lacking any interface or interaction that supports SRL during the forethought phase. League of Legends itself provides little guidance on how to practice effectively, meaning that players must turn to external resources. Existing literature acknowledges this phenomenon of players seeking out external resources (Taylor, 2006; Consalvo, 2009), and it is likely that there is a connection between SRL processes and skills and one's ability to seek out the correct resources. It may be that most novice players have not sought these resources and therefore have not developed the same SRL skills for the forethought phase as their more skilled counterparts. Also worth noting is that most of the third-party tools that exist for League of Legends do not aim to help players with goal setting or training routines. Coaches are ultimately the best resource in this area, but non-expert and novice players are less likely to have access to coaches than expert players, resulting in significant differences. This may be a point of concern, as uncertainty in how to gain skill or set realistic goals could result in frustration and discontinuation of play (Brusso et al., 2012; Esteves et al., 2021), leading to higher churn rates.

It is also necessary to acknowledge that the differences between the results of this study and the results of Kitsantas and Zimmerman (2002)'s study are likely also influenced by the nature of the game and what it means to be a novice of that game. League of Legends requires players to complete a tutorial before beginning play, meaning that complete beginners, and certainly novices, possess some basic knowledge of gameplay and terminology. By contrast, volleyball novices recruited from a public court, such as those in Kitsantas and Zimmerman (2002)'s study, may or may not have ever looked at any formal documentation on how to play. While this does not necessarily make one game easier than the other, it does suggest an inherent difference in the knowledge level of novice players, which may explain the lack of statistical difference in knowledge of last hitting.

### Implications

We identify three main areas where our findings have noteworthy implications for future research and development, which we discuss below. We also note that our findings and the implications we discuss in this section may not be unique to esports and may apply to digital games at large. We hope to explore this further in future work.

#### Computational Support for Esports

The first area in which these results have implications is in the domain of computational support for esports. A great number of computational tools for esports exist, which broadly provide players with assistance in decision making (Chen et al., 2018a,b; Christiansen et al., 2019; Eger and Sauma Chacón, 2020) and review of gameplay (Wallner and Kriglstein, 2016, 2020; Kuan et al., 2017; Afonso et al., 2019). These tools are often explicitly motivated by the desire to help players learn and master their game. Based on our results, future tools can be designed with SRL in mind. Specifically, we see that novices differ greatly in their execution of SRL processes in the forethought phase compared to non-experts and experts. Thus, a targeted and effective way to assist novices through computational support may be through the development of tools that directly address this specific phase. Specifically, tools that can help novices with goal setting and practice regimens may be beneficial, especially since previous work has shown that unrealistic goals can lead to worse performance (Brusso et al., 2012). While computational tools designed for the other phases may also be helpful to players, the lack of statistically significant differences in SRL processes in these phases indicates that players, especially novice players, may not need additional support in these areas.

#### Transferring SRL Skills From Esports to Academics

The second implication of these results is that esports may be an engaging way to train SRL skills that can transfer to academic contexts. In the learning literature, transfer is defined as the extent to which a student can apply a learned knowledge or skill to new situations different from the learning context (Perkins and Salomon, 1992). The literature also defines two types of transfer: near transfer, referring to transfer occurring in a new situation that resembles the learning context where the knowledge or skill is learned, and far transfer, referring to transfer occurring in situations where the learning context is very different (Schuster et al., 2020). So far, transfer of SRL from games to academics has predominantly focused on educational games (Nietfeld, 2017), which are primarily examples of near transfer. By contrast, transferring SRL from esports to academic contexts would be an example of far transfer. Previous work on SRL transfer suggests that far transfer is often less successful (Dignath and Veenman, 2021). For example, Raaijmakers et al. (2018) found that SRL skills taught in biology did not transfer to math, which they considered an example of far transfer.

Despite this evidence, however, there have been arguments in favor of far transfer. McCardle (2015a) argues that much of the work on transfer looks at what the students learn rather than how they learn, and suggests that how they learn, which is a critical component of SRL, would successfully transfer even in circumstances where what they learn does not. This argument aligns with the defining traits of far transfer from Schuster et al. (2020), discussed above. This argument is further supported by previous work that found that athletes who engage SRL skills within their sport tend to engage the same skills in academic contexts. For example, in a case study of a table tennis player who was also a university student, McCardle (2015b) found that the athlete leveraged the same SRL skills, such as task-understanding and goal setting, in both contexts. Previous work on the academic impact of esports highlighted similar trends to McCardle (2015b)'s case study. For example, students who were interviewed by Cho et al. (2019) explicitly stated that they would take the skills and logic they used to communicate or make decisions in-game and use them in the classroom. These findings from previous work are encouraging and suggest that SRL skills may also successfully far transfer to academic contexts.

While esports for SRL training would not necessarily train specific cognitive strategies that could directly transfer to academic contexts, they may be able to provide students with an opportunity to develop more general meta-cognitive skills, providing them with a foundation upon which academic context-specific strategies can then be built. As White and Frederiksen (2005) demonstrate, developing strong meta-cognitive knowledge is beneficial to academic performance and as Bartolomé and Steffens (2011) argue, these skills do transfer across domains. As esports can be played at home and in the absence of a teacher, they may provide students with more opportunities to practice using meta-cognitive skills. Further, since esports are engaging, and, as demonstrated by our results, players at all skill levels are highly motivated to succeed, getting students to play would likely not be difficult. In future work, we hope to explore this further and empirically examine the phenomenon of transfer of SRL between esports and academics.

#### Data-Driven and E-Learning

The third area where these results have implications is data-driven and e-learning environments, where data is used to motivate, encourage, and assist students. These environments may be able to help promote SRL in students by emulating esports-style interfaces within their applications. The results of our study suggested that the presence of League of Legends' in-game UI, which tracks gold, experience, kills, and creep score among other points, promoted SRL in the performance phase. Similarly, the post-game statistics screens, which display aggregate counts of all players' performances, promoted SRL in the reflection phase. Using this design approach as inspiration, e-learning applications, especially those that take a gamification approach, could potentially support the engagement of SRL skills in students by developing similar UI elements for their applications. Some applications have already begun to explore this possibility space through Open Learner Models (OLMs), which are conceptually similar to these UIs, in that they display measures and evaluations of a students' performance. OLMs have demonstrated great promise in supporting SRL in learning contexts (Hooshyar et al., 2020). Future work can explore opportunities to use esports and esports UIs to expand, and even gamify, open learner model design and support SRL and learning in more engaging and motivating ways. This suggestion is in line with observations made by previous work on personal informatics that have turned to games to identify opportunities for developing more engaging data visualization systems (Kou and Gui, 2018; Rapp, 2018).

These results also have implications for the implementation of Learning Analytics Dashboards (LADs) (Bodily and Verbert, 2017; Bodily et al., 2018). While many LADs seek to motivate and aid students through comparison of their own progress against that of their peers (Fritz, 2011; Santos et al., 2013; Park and Jo, 2015), previous work also found that such comparison is often undesired by students or can have the opposite effect, either de-motivating them or making it harder for them to set goals and follow plans (Reimers et al., 2015; Aguilar, 2016; Rets et al., 2021). Similar to these LADs, League of Legends' UI elements provide players with critical information about their performance, which includes comparisons with other players, most notably post-game. The results of our study suggest that the post-game comparison does not have detrimental effects on students' motivation, and LADs may be able to leverage design insights from esports UI's in order to overcome this challenge. Most notably, presenting comparative information only at the end of the term, the academic equivalent of ”post-game”, may provide students with valuable information that can motivate a desire to overcome shortcomings in the following term.

## Limitations and Future Work

We acknowledge the sample size as a limitation of this work. We chose to replicate the sample size of the original study to ensure that the results of this work could be directly compared to the original results, and claims about how SRL compares between traditional athletics and esports could be made. However, we recognize that a larger sample size may reveal more significant differences between expertise levels.

Further, like the original study, we do not question players about their experience with other games. This may result in inherent differences between players in a given expertise level, i.e., those who have played another game before may perform better than those for whom League of Legends is their first esport. We hope to address this limitation through further exploration of this topic in future work.

We also acknowledge that we have only looked at a single esport game, and that other games may present different findings. This may be especially true if our assumption that League of Legend's in-game elements are teaching players SRL skills is correct. While we argue that these results are likely generalizable given that many esports games, including DotA2 and Heroes of the Storm, follow a similar design framework, we acknowledge that future work is necessary to ensure the generalizability of the results.

With this in mind, we present this work as an exploratory first look at SRL in an esports context, how it differs from traditional sports, and the implications of SRL's manifestation within esports. We hope to follow it with a larger scale study in future work. We also hope to expand the study of SRL in esports to look at individual processes and phases as well as SRL in the context of other games and models. Further, in future work, we hope to explore and expand upon the three implications we discussed in the previous section.

## Conclusion

In this work, we replicated Kitsantas and Zimmerman (2002)'s micro-analytic study in the context of League of Legends, in order to examine differences in SRL processes between expert, non-expert, and novice players. Our results found that there were significant differences in the forethought phase, but none in the performance and self-reflection phases. We discuss that these findings, which are different from those of the original study, may be the result of the design of the game, and suggest that League of Legends, and similar esport games, may be training players in SRL skills. Based on these conclusions, we identify opportunities for computational support for esports, suggest that esports may be a potential avenue for training SRL skills, and suggest ways in which data-driven and e-learning environments may be able to learn from esports to improve learning and SRL skill acquisition. In future work, we hope to explore these three implications and expand on our understanding of SRL in esports.

## Data Availability Statement

The raw data supporting the conclusions of this article can be made available by the authors upon request.

## Ethics Statement

The studies involving human participants were reviewed and approved by UC Santa Cruz Office of Research. The patients/participants provided their written informed consent to participate in this study.

## Author Contributions

EK led the study, conducted the interviews, collected and analyzed the data, and wrote the manuscript. CG acted as the second researcher for the qualitative analysis and assisted in the development and writing of the discussion. MS was the PI on the project and EK's Ph.D. advisor, offering feedback throughout the study, and providing guidance in writing the manuscript. All authors contributed to the article and approved the submitted version.

## Funding

This work was partially supported by the National Science Foundation under grant no. 1917855.

## Conflict of Interest

The authors declare that the research was conducted in the absence of any commercial or financial relationships that could be construed as a potential conflict of interest.

## Publisher's Note

All claims expressed in this article are solely those of the authors and do not necessarily represent those of their affiliated organizations, or those of the publisher, the editors and the reviewers. Any product that may be evaluated in this article, or claim that may be made by its manufacturer, is not guaranteed or endorsed by the publisher.
